# Supplementary material for: Sex Difference and Smoking Effect of Lung Cancer Incidence in Asian Population
Source: Cancers (Basel). 2020 Dec 31;13(1):113. doi: 10.3390/cancers13010113 (PMC7794680; doi:10.3390/cancers13010113)
Supplement: Supplementary file 1 [file cancers-13-00113-s001.pdf]

Supplementary Materials: Sex Difference and Smoking Effect of Lung Cancer Incidence in Asian Population

Boyoung Park, Yeol Kim, Jaeho Lee, Nayoung Lee and Seung Hun Jang

Table S1. Adjusted risk of lung cancer according to the measures of various smoking statuses.

Smoking Measures	Male			Female			Sex-Smoking Interaction
	N	Adjusted HR (95% CI) [1]	*P*-Value	*N*	Adjusted HR (95% CI) [1]	*P*-Value	
Smoking status							
Never	1,157,106	2.41 (2.34–2.48)	<0.001	4,010,591	1.00 (reference)	-	-
Former	513,760	2.95 (2.84–3.07)	<0.001	26,111	1.45 (1.16–1.81)	<0.001	0.261
Current	777,070	6.27 (6.08–6.47)	<0.001	84,506	2.70 (2.48–2.94)	<0.001	-
Duration of smoking							
1–9 years	53,677	1.52 (1.06–2.18)	0.022	25,164	1.00 (reference)	-	-
10–19 years	186,724	1.44 (1.03–2.01)	0.035	34,884	1.35 (0.92–1.99)	0.127	-
20–29 years	475,970	2.25 (1.62–3.12)	<0.001	29,250	1.87 (1.31–2.69)	<0.001	<0.001
≥30 years	574,023	4.71 (3.41–6.52)	<0.001	21,269	2.46 (1.75–3.45)	<0.001	-
Duration of former smoking							
1–9 years	413,86	3.02 (1.23–7.43)	0.016	9643	1.00 (reference)	-	-
10–19 years	129,515	3.28 (1.35–7.94)	0.009	8331	2.24 (0.80–6.27)	0.126	-
20–29 years	169,258	4.59 (1.90–11.07)	<0.001	4849	4.73 (1.82–12.29)	0.001	0.017
≥30 years	173,471	8.53 (3.54–20.54)	<0.001	3265	4.21 (1.64–10.79)	0.003	-
Duration of current smoking							
1–9 years	12,291	2.09 (1.37–3.19)	0.001	15,521	1.00 (reference)	-	-
10–19 years	57,209	1.68 (1.14–2.46)	0.009	26,553	1.11 (0.91–1.97)	0.623	-
20–29 years	306,712	2.25 (1.58–3.20)	<0.001	24,401	1.34 (1.21–2.50)	0.144	0.019
≥30 years	400,552	3.82 (2.70–5.42)	<0.001	18,004	1.74 (1.37–3.19)	0.003	-
Quantity of smoking							
1–10 cigarettes/day	108,145	1.60 (1.36–1.88)	<0.001	39,841	1.00 (reference)	-	-
11–20 cigarettes/day	491,187	2.23 (1.92–2.59)	<0.001	49,727	1.37 (1.14–1.65)	<0.001	0.143
>20 cigarettes/day	691,498	3.24 (2.79–3.75)	<0.001	21,049	1.67 (1.35–2.07)	<0.001	-
Quantity of smoking in former smokers							
1–10 cigarettes/day	48,946	1.50 (0.97–2.34)	0.070	11,361	1.00 (reference)	-	-
11–20 cigarettes/day	200,661	2.15 (1.41–3.28)	<0.001	10,334	1.55 (0.92–2.64)	0.101	0.180
>20 cigarettes/day	264,153	3.27 (2.15–4.97)	<0.001	4416	1.41 (0.76–2.60)	0.273	-
Quantity of smoking in current smokers							
1–10 cigarettes/day	59,199	1.87 (1.57–2.22)	<0.001	28,480	1.00 (reference)	-	-
11–20 cigarettes/day	290,526	2.66 (2.26–3.12)	<0.001	39,393	1.35 (1.11–1.64)	0.003	0.208
>20 cigarettes/day	427,345	4.05 (3.46–4.75)	<0.001	16,633	1.77 (1.41–2.22)	<0.001	-

Pack-year							
0–9	186,132	1.37 (1.16–1.61)	<0.001	64,184	1.00 (reference)	-	-
10–19	362,880	1.91 (1.64–2.23)	<0.001	28,880	1.57 (1.29–1.91)	<0.001	-
20–29	345,016	2.61 (2.25–3.04)	<0.001	10,844	1.68 (1.33–2.12)	<0.001	0.007
≥30	396,802	3.99 (3.44–4.63)	<0.001	6709	2.24 (1.78–2.81)	<0.001	
Pack-year in former smokers							
0–9	118,093	1.69 (1.13–2.55)	0.011	18,433	1.00 (reference)	-	-
10–19	159,305	2.10 (1.41–3.13)	<0.001	4817	2.14 (1.24–3.69)	0.006	-
20–29	109,053	2.97 (1.99–4.43)	<0.001	1656	2.79 (1.47–5.29)	0.002	0.026
≥30	127,309	4.76 (3.21–7.07)	<0.001	1205	1.85 (0.91–3.76)	0.090	-
Pack-year in current smokers							
0–9	68,039	1.86 (1.54–2.24)	<0.001	45,751	1.00 (reference)	-	-
10–19	203,575	2.30 (1.95–2.71)	<0.001	24,063	1.41 (1.14–1.74)	0.001	-
20–29	235,963	2.79 (2.37–3.29)	<0.001	9188	1.46 (1.14–1.88)	0.003	0.379
≥30	269,493	4.26 (3.63–5.00)	<0.001	5504	2.16 (1.70–2.75)	<0.001	-
Years since cessation in former smokers							
Current smoker	777,070	2.43 (2.23–2.64)	<0.001	84,506	1.00 (reference)	-	-
0–4	156,560	1.67 (1.52–1.85)	<0.001	11,981	0.65 (0.47–0.90)	0.009	0.469
≥5	357,200	0.92 (0.84–1.01)	0.066	14,130	0.46 (0.33–0.64)	<0.001	-

N, number; HR, hazard ratio; CI, confidence interval. [1] Adjusted for age, alcohol consumption, physical activity, body mass index, family history of cancer, and medical history of lung diseases including emphysema, chronic pulmonary obstructive disease, pneumoconiosis, and interstitial pulmonary disease.

Table S2. Adjusted risk of lung cancer according to the combination of smoking quantity and duration of smoking abstinence with female current smokers as reference.

	**PkY**	**Years Since Cessation**	**Male**			**Female**		
			N	Adjusted HR (95% CI) [1]	*P*-value	*N*	Adjusted HR (95% CI) [1]	*P*-value
Current	-	-	1,157,106	2.42 (2.22–2.63)	<0.001	4,010,591	1.00 (reference)	-
Past	<10	<5	20,997	0.82 (0.62–1.09)	0.172	7845	0.38 (0.20–0.71)	0.003
-	-	≥5	97,096	0.57 (0.49–0.66)	<0.001	10,588	0.33 (0.20–0.55)	<0.001
-	<20	<5	44,599	1.14 (0.96–1.35)	0.138	2530	0.85 (0.51–1.41)	0.524
-	-	≥5	114,706	0.64 (0.56–0.72)	<0.001	2287	0.60 (0.34–1.06)	0.078
-	<30	<5	39,048	1.38 (1.20–1.49)	<0.001	920	0.99 (0.49–1.99)	0.973
-	-	≥5	70,005	0.89 (0.79–1.01)	0.070	736	0.84 (0.40–1.78)	0.652
-	≥30	<5	51,916	2.09 (1.88–2.33)	<0.001	686	0.75 (0.36–1.58)	0.450
-	-	≥5	75,393	1.34 (1.21–1.49)	<0.001	519	0.44 (0.17–1.19)	0.105

PkY, pack-years; N, number; HR, hazard ratio; CI, confidence interval. [1]Adjusted for age, alcohol consumption, physical activity, body mass index, family history of cancer, and medical history of lung diseases including emphysema, chronic pulmonary obstructive disease, pneumoconiosis, and interstitial pulmonary disease.
